# Statistical non-independence of brain metabolite concentrations whether normalized to creatine or water

**DOI:** 10.1177/0271678X241290018

**Published:** 2024-10-18

**Authors:** Richard J Maddock

**Affiliations:** Department of Psychiatry and Behavioral Sciences, and Imaging Research Center, University of California Davis, Sacramento, CA, USA

**Keywords:** Absolute values, magnetic resonance spectroscopy, metabolic coupling, glutamate, partial volume

## Abstract

1H-MRS investigators studying brain metabolite concentrations often attribute biological significance to correlations between calculated metabolite values within the same voxel. A recent report in this journal provides a valuable perspective on how statistical non-independence of such values can undermine biological interpretations of their correlations. However, careful examination of this issue suggests their critical analysis does not go far enough. Hong et al. claim that appropriate water normalization, unlike creatine normalization, eliminates the problem of spurious correlation. Both logical and empirical considerations show this is not the case. Correlations between water-normalized metabolite values are also prone to substantial spurious correlations.

It has become increasingly common for investigators conducting 1H-MRS studies to attribute biological significance to correlations between brain metabolite concentrations estimated in the same voxel^1–3^, especially positive correlations between NAA and glutamate. While shared metabolic pathways or a common underlying neurobiological cause can lead to correlations between concentrations of two brain metabolites, computational factors can also cause such correlations. The recent paper in this journal by Hong et al.^4^ provides a valuable perspective on two of the most important computational factors that confound biological interpretations of statistical associations between metabolites – Pearson’s spurious correlation between ratios with the same denominator and spectral correlation due to overlapping resonances. Hong et al.^4^ propose methods for eliminating both sources of statistical non-independence between metabolite values. Using their methods, they observed no significant correlation between NAA and glutamate in healthy volunteers and argue against a model of neurometabolic coupling between NAA and glutamate. The authors have provided a valuable analysis of the problems that plague attempts to interpret correlations between metabolites. However, a few logical errors in their paper undermine some of their conclusions and may mislead other investigators.

The most significant problem pertains to the claim that water-normalization effectively eliminates Pearson’s spurious correlation between metabolite ratios with the same denominator. A second problem is that the authors have not estimated NAA independently from NAAG in their *in vivo* dataset. While their finding of no evidence to support a neurometabolic coupling between NAA and glutamate remains valid, their proposal that water normalization eliminates spurious correlations is invalid for human studies.

In considering the problem of spurious correlation for metabolite/creatine and metabolite/water ratios, the authors provide Pearson’s equation^5^ demonstrating the key role of the coefficients of variation (V) of the numerators and denominators inherent in all metabolite quantification methods. Hong et al. correctly emphasize the point that for correlations between metabolite X and metabolite Y, normalized as X/Z and Y/Z, the problem of spurious correlation becomes negligible when V_Z_ ≪ V_X_ and V_Y_. They further state “*due to the very high signal-to-noise ratio of the unsuppressed water, the inequality V_water_ ≪ V_NAA_, V_glutamate_ holds. Based on equation (3), Pearson’s spurious correlation of ratios for glutamate and NAA can be eliminated by using [glutamate]/[water] and [NAA]/[water] instead of [glutamate]/[tCr] and [NAA]/[tCr].*” In their subsequent Monte Carlo analysis, they rely on the assumption that V_water_ ≪ V_metabolite_ in generating the simulated data. Accordingly, they find negligible spurious correlation in their simulated water-normalized NAA and glutamate data.

While the above reasoning is valid for water-normalized metabolites acquired from a phantom, it does not apply to water-normalized metabolite values acquired *in vivo*. Specifically, the inequality V_water_ ≪ V_metabolite_ is true for phantom experiments but is decidedly not true when the water signal requires correction for partial volume and relaxation effects, as is the case for *in vivo* experiments. The most significant source of nuisance variance in the corrected water signal is probably noise in the estimation of voxel segmentation fractions. Other sources of added noise may include individual subject deviations from canonical tissue water content and relaxation rates and variance due to head displacement between the high resolution scan, the water suppressed MRS scan, and the water non-suppressed MRS scan.

Evidence against their claim that water normalization eliminates spurious correlations can be found in the authors’ own *in vivo* data. For metabolites in the occipital cortex and in the medial prefrontal cortex, the authors report the partial correlation coefficients between all water-normalized metabolite estimates (“absolute concentrations”) after controlling for age, gray matter and white matter fractions in the voxel. Five of the reported metabolites are generally considered well-measured in 3 Tesla human studies – NAA, total creatine, total choline compounds, glutamate, and myo-inositol. GABA, glutamine, glutathione and NAAG are also reported, but these are less precisely measured with the scanning parameters used by the authors. Taking only the ten pair-wise correlations between the five well-measured metabolites in each voxel, we can use Fisher’s *r* to *z* conversion to calculate the mean and median *r* values in the authors’ reported data. For the medial prefrontal cortex, the mean and median *r* values are both 0.67, for the occipital cortex they are 0.32 and 0.38, and for all 20 correlations across both voxels, they are 0.51 and 0.56 respectively. The overall central tendency of these correlations approximating *r = *0.5 is what one expects when the true correlations between metabolites are negligible (approximating *r = *0.0), and the coefficient of variation of the corrected water value in the denominator is approximately equal to coefficients of variation of the metabolites in the numerators^4–6^. Thus, the authors’ *in vivo* data invalidates their working assumption that V_water_ ≪ V_metabolites_. Relatedly, coefficients of variation for GABA^7^ and glutamate^8^ in large studies are consistently greater when normalizing to water than when normalizing to creatine, an unexpected finding if V_water_ ≪ V_creatine_. I encourage other MRS investigators to examine their own datasets for similar signs of statistical non-independence in both water- and creatine-normalized data before interpreting any individual correlations as biologically meaningful.

A second concern pertains to the authors’ LCModel estimates of NAA and NAAG. Although they report imposing a soft constraint to set the values of NAA and NAAG to a ratio of 10:1, the data reveal that a hard constraint was imposed. Tables 1 and 2 in Hong et al.^4^ show a perfect correlation (r = 1.0) between NAA and NAAG values in each voxel. This is biologically implausible but would occur if the NAAG value was always set to exactly 10% of the NAA value. Consequently, their NAA and NAAG values contain the same information and are likely proportional to the estimate of NAA plus NAAG.
